# Supplementary material for: Soluble uric acid suppresses neutrophil-mediated host defense in sepsis
Source: Nat Commun. 2026 May 19;17:4453. doi: 10.1038/s41467-026-73090-4 (PMC13187337; doi:10.1038/s41467-026-73090-4)
Supplement: Supplementary file 1 — Supplementary information [file 41467_2026_73090_MOESM1_ESM.pdf]

Supplementary information

Supplementary Tables

Supplementary Table S1: Murine and human primer sequences.

**Mouse Genes**		**Primer sequences**
KIM-1	Forward	5‘-TCAGCTCGGGAATGCACAA-3‘
	Reverse	5‘-TGGTTGCCTTCCGTGTCTCT-3‘
Tnfα	Forward	5‘-CCACCACGCTCTTCTGTCTAC-3‘
	Reverse	5‘-AGGGTCTGGGCCATAGAACT-3‘
Il6	Forward	5‘-TGATGCACTTGCAGAAAACA-3‘
	Reverse	5‘-ACCAGAGGAAATTTTCAATAGGC-3‘
Nlrp3	Forward	5‘-CCACAGTGTAACTTGCAGAAGC-3‘
	Reverse	5‘-GGTGTGTGAAGTTCTGGTTGG-3‘
Fibronectin 1	Forward	5‘-GGAGTGGCACTGTCAACCTC-3‘
	Reverse	5‘-ACTGGATGGGGTGGGAAT-3‘
18s RNA	Forward	5‘-GCAATTATTCCCCATGAACG-3‘
	Reverse	5‘-AGGGCCTCACTAAACCATCC-3‘
**Human Genes**		**Primer sequences**
XDH	Forward	5‘-CTCAGTCAGCCTCTCGCCAT-3‘
	Reverse	5‘-TATCCACGTCACACGCTCCC-3‘
Cybb	Forward	5‘-CTCTGAACTTGGAGACAGGCAAA-3‘
	Reverse	5‘-CACAGCGTGATGACAACTCCAG-3‘
TLR4	Forward	5‘-CTTCTCCTTCCTGATCGTGG-3‘
	Reverse	5‘-GCTGGTTATCTCTCAGCTCCA-3‘
P38a	Forward	5‘-GCAGCACTACTTCTTGACCACC-3‘
	Reverse	5‘-TCTGCTCCTGAGCATTGACGTC-3‘
JNK1	Forward	5‘-GCTCTTGGAGAAGCACAACGAG-3‘
	Reverse	5‘-ACACCAGGCGGATGCTCCTCT-3‘
GAPDH	Forward	5‘-GTCTCCTCTGACTTCAACAGCG-3‘
	Reverse	5‘-ACCACCCTGTTGCTGTAGCCAA-3‘

Supplementary Figures

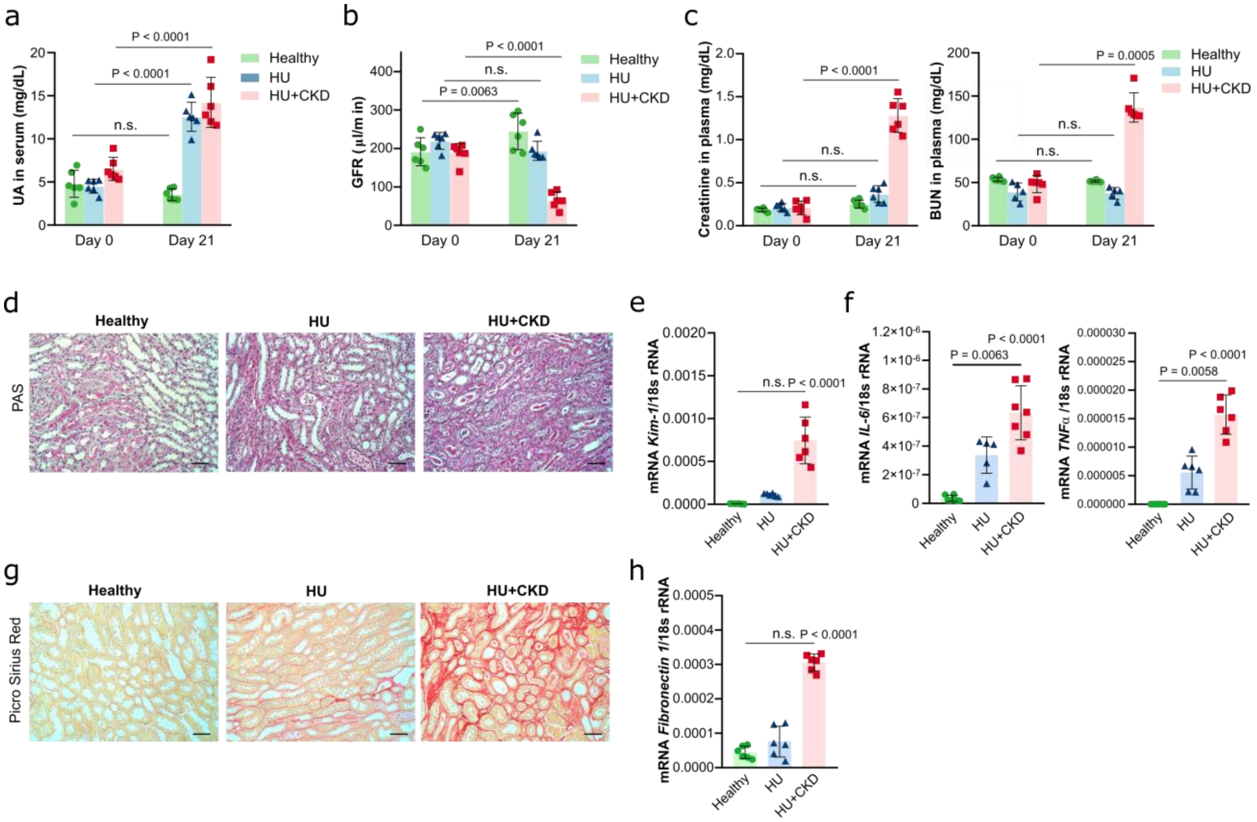

Supplemental Figure S1: Baseline characteristics of the mouse model of hyperuricemia with or without CKD. Alb-creERT2;*Glut9*$^{lox/lox}$ mice and *Glut9*$^{lox/lox}$ control mice were injected intraperitoneally with tamoxifen. Mice were fed either an acidogenic diet enriched with inosine or a standard chow diet with inosine for 24 days. (**a** to **c**) Serum uric acid (UA) (a), glomerular filtration rate (GFR) (b), plasma creatinine and blood urea nitrogen (BUN) (c) levels of *Glut9*$^{lox/lox}$ mice with chow diet with inosine (healthy), Alb-creERT2;*Glut9*$^{lox/lox}$ mice with chow diet with inosine (hyperuricemia, HU), or acidogenic diet with inosine (hyperuricemia with chronic kidney disease, HU+CKD) and on day 21. (a-c, n = 6, one technical replicate of 6 biological replicates for each group). (**d** and **e**) Periodic acid-Schiff (PAS) staining (20x magnification, scale bar 20 µm) (d) and intrarenal mRNA expression levels of kidney injury maker *Kim-1* (e) illustrating tubular injury in hyperuricemic CKD mice but not in healthy and HU mice on day 24 (e, n = 6, one technical replicate of 6 biological replicates for each group). (**f**) Intrarenal mRNA expression levels of the inflammatory marker *Il-6* and tumor necrosis factor α (*Tnfα*) (n = 6, one technical replicate of 6 biological replicates for each group). (**g** and **h**) Picro Sirius red staining (20x magnification, scale bar 20 µm) (g) and intrarenal mRNA expression levels of the fibrosis maker *Fibronectin-1* illustrating interstitial fibrosis in hyperuricemic CKD mice but not in healthy and HU mice on day 24 (n = 6, one technical replicate of 6 biological replicates for each group). Data are mean ± SD. P values are determined by one- or two-way ANOVA. n.s., not significant. Source data for a-c, e-f, and h are provided as a Source Data file.

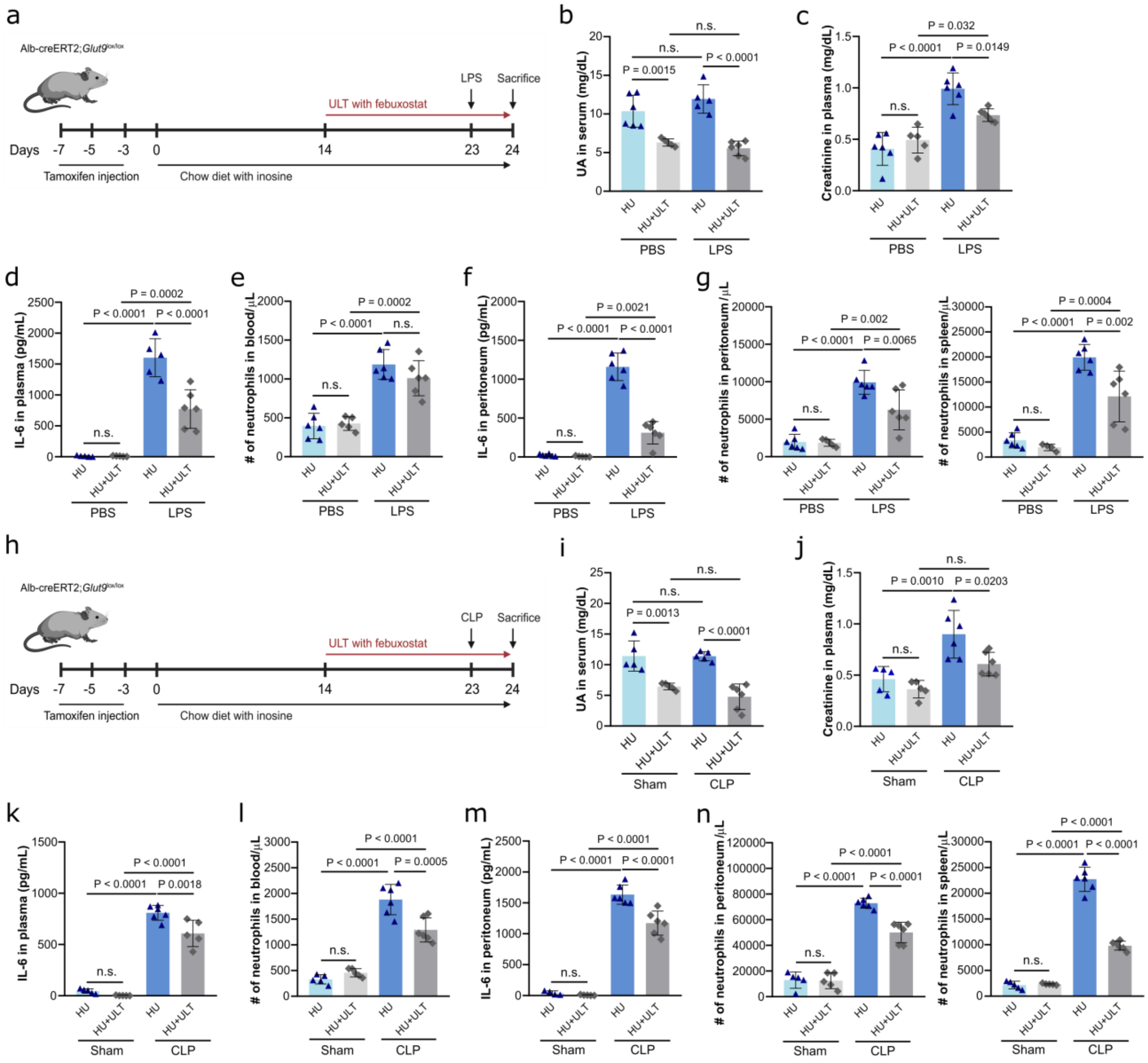

Supplemental Figure S2: Febuxostat treatment improves outcomes in hyperuricemic mice following peptidoglycan-induced gram-positive and bacterial sepsis in mice. (**a**) Alb-creERT2;*Glut9*$^{lox/lox}$ mice were injected intraperitoneally with tamoxifen and fed an inosine-rich diet for 24 days. Urate-lowering therapy (ULT) with febuxostat was initiated on day 14 to lower serum UA levels. On day 23, mice were randomized and received either an injection of LPS or PBS and were sacrificed 24 hours later. Created in BioRender. Steiger, S. (2026). https://BioRender.com/naodono. (**b** and **c**) Serum UA (b) and plasma creatinine (c) levels of hyperuricemic (HU) mice after LPS or PBS injection on day 23 and 24. (b-c, n = 5-6, one technical replicate of 5-6 biological replicates for each group). (**d** and **f**) Concentrations of IL-6 measured in plasma (d) and peritoneum (f) from HU mice with and without febuxostat after LPS or PBS injection via ELISA on day 24 (d-f, n = 5-6, one technical replicate of 5-6 biological replicates for each group). (**e** and **g**) Number (#) of neutrophils (CD45+CD11b+Ly6G+Ly6C-) in blood (e), peritoneum and spleen (g) per µL determined by flow cytometry on day 24 (e-g, n = 6, one

technical replicate of 6 biological replicates for each group). (**h**) In hyperuricemic (HU) mice, urate-lowering therapy (ULT) with febuxostat was initiated on day 14. On day 23, mice unwent either cecal ligation and puncture (CLP) or sham surgery and were sacrificed 24 hours later. Created in BioRender. Steiger, S. (2026). https://BioRender.com/8wc9qt7. (**i** and **j**) Serum UA (i) and plasma creatinine (j) levels from HU mice with or without febuxostat after sham or CLP surgery on day 24 (i-j, n = 5-6, one technical replicate of 5-6 biological replicates for each group). (**k** and **m**) Concentrations of IL-6 measured in plasma (k) and peritoneum (m) via ELISA (k-m, n = 5-6, one technical replicate of 5-6 biological replicates for each group). (**l** and **n**) Number (#) of neutrophils (CD45+CD11b+Ly6G+Ly6C-) in blood (l), peritoneum and spleen (n) per µL from hyperuricemic (HU) mice with or without febuxostat after sham or CLP surgery determined by flow cytometry (l-n, n = 5-6, one technical replicate of 5-6 biological replicates for each group). Data are mean ± SD. P values are determined by one-way ANOVA. n.s., not significant. Source data for b-g and i-n are provided as a Source Data file.

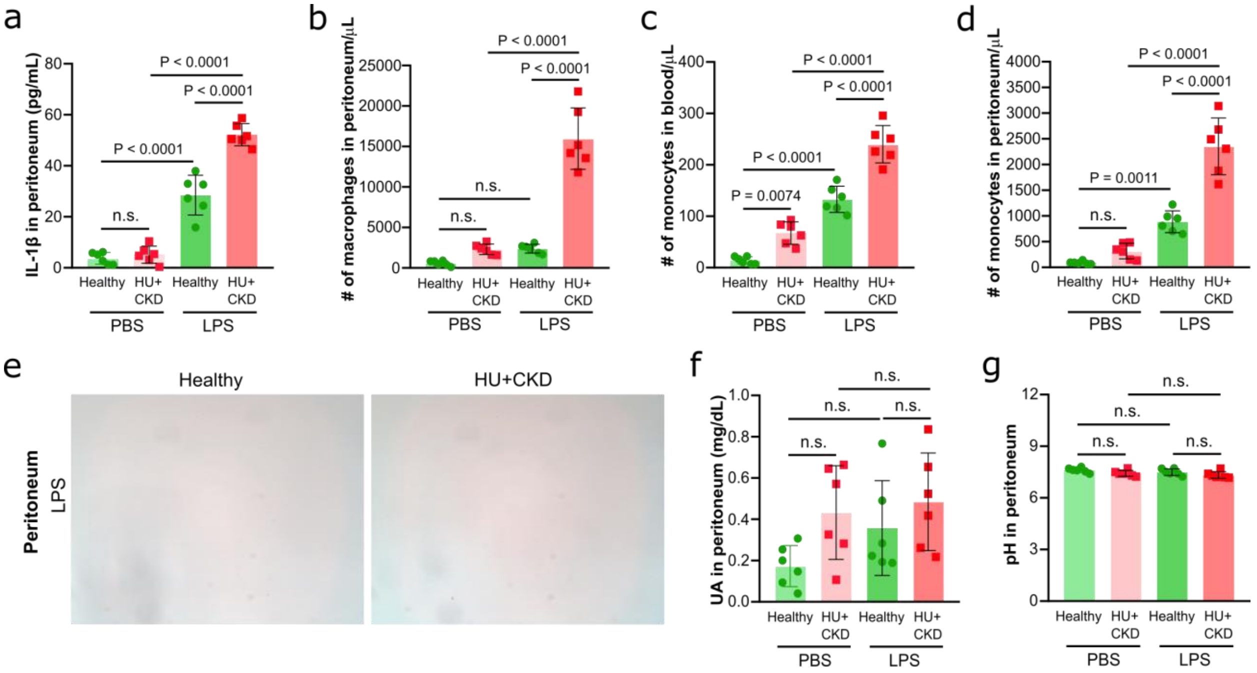

Supplemental Figure S3: UA crystals are not responsible for inducing inflammation in the peritoneum during LPS-induced endotoxemia. Alb-creERT2;*Glut9*$^{lox/lox}$ mice and *Glut9*$^{lox/lox}$ control mice were injected intraperitoneally with tamoxifen. Both groups were fed either an acidogenic diet with inosine (HU+CKD) or a chow inosine-rich (Healthy) diet for 24 days. On day 23, mice received an injection of LPS or PBS into the peritoneal cavity and were sacrificed 24 hours later. (**a**) Concentrations of IL-1β measured in plasma from healthy and hyperuricemic CKD (HU+CKD) mice after LPS or PBS injection via ELISA on day 24 (n = 6, one technical replicate of 6 biological replicates for each group). (**b** - **d**) Number (#) of macrophages in peritoneum (b), and monocytes in blood (c) and peritoneum (d) per mL from healthy and hyperuricemic CKD mice after LPS or PBS injection on day 24 determined by flow cytometry. (b-d, n = 6, one technical replicate of 6 biological replicates for each group). (**e**) Representative images of peritoneal wash in healthy and hyperuricemic CKD mice after LPS injection (original magnification 20x). (**f** and **g**) Peritoneum UA levels (f) and pH (g) in healthy and hyperuricemic CKD mice after LPS or PBS injection on day 24. (f-g, n = 6, one technical replicate of 6 biological replicates for each group). Data are mean ± SD. P values are determined by one-way ANOVA. n.s., not significant. Source data for a-d and f-g are provided as a Source Data file.

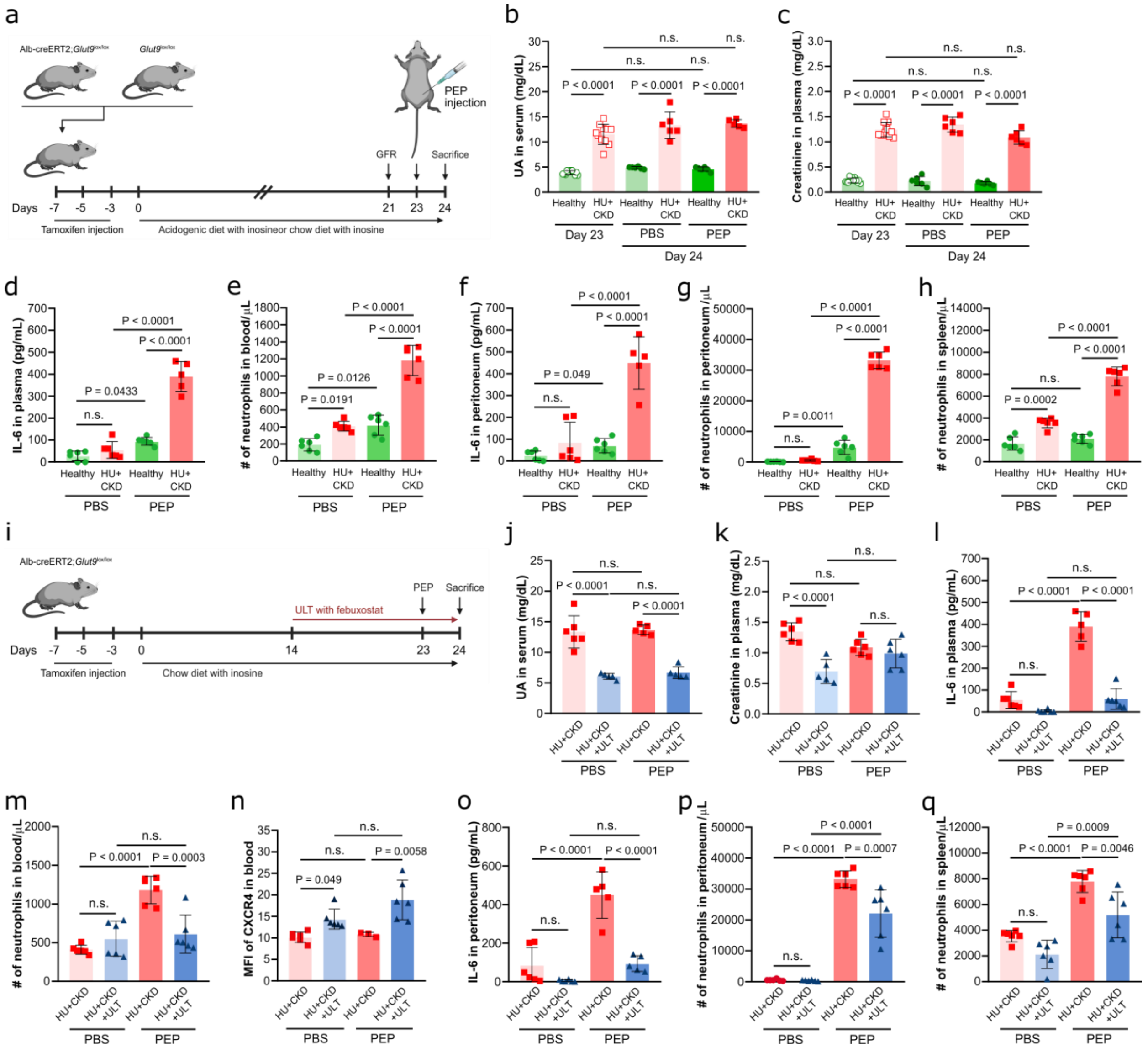

Supplemental Figure S4: CKD-related HU aggravates the immune response to peptidoglycan-induced inflammation in mice. (**a**) Alb-creERT2;*Glut9*$^{lox/lox}$ mice and *Glut9*$^{lox/lox}$ control mice were injected intraperitoneally with tamoxifen. Both groups were fed either an acidogenic diet with inosine or an inosine-rich diet for 24 days. On day 23, mice were randomized and received either an injection of peptidoglycan (PEP) or PBS into the peritoneal cavity, and were sacrificed 24 hours later. Created in BioRender. Steiger, S. (2026). https://BioRender.com/vtfkn7k. (**b** and **c**) Serum UA (b) and plasma creatinine (c) levels of healthy and hyperuricemic CKD (HU+CKD) mice after PEP or PBS injection on day 23 (n = 10-11) and 24 (n = 5-6). (b-c, n = 5-11, one technical replicate of 5-11 biological replicates for each group). (**d** and **f**) Concentrations of IL-6 measured in plasma (d) and peritoneum (f) via ELISA on day 24. (d-f, n = 5-6, one technical replicate of 5-6 biological replicates for each group). (**e**, **g** and **h**) Number (#) of neutrophils (CD45+CD11b+Ly6G+Ly6C-) in blood (e), peritoneum (g) and spleen (h) per mL determined by flow cytometry on day 24. (e-h, n = 5-6, one technical replicate of 5-6 biological replicates for each group). (**i**) Mice were assigned

to healthy and HU+CKD groups as previously described, and urate-lowering therapy (ULT) with febuxostat was initiated on day 14. On day 23, mice received an injection of PEP or PBS, and were sacrificed 24 hours later. Created in BioRender. Steiger, S. (2026). https://BioRender.com/5no42bc. (**j** and **k**) Serum UA (j) and plasma creatinine (k) levels from HU+CKD mice with or without febuxostat after PEP or PBS injection on day 24. (j-k, n = 5-6, one technical replicate of 5-6 biological replicates for each group). (**l** and **o**) Concentrations of IL-6 measured in plasma (l) and peritoneum (o) via ELISA. (l-o, n = 5-6, one technical replicate of 5-6 biological replicates for each group). (**m**, **p** and **q**) Number (#) of neutrophils (CD45+CD11b+Ly6G+Ly6C-) in blood (m), peritoneum (p) and spleen (q) per mL determined by flow cytometry. (m-q, n = 5-6, one technical replicate of 5-6 biological replicates for each group). (**n**) MFI of CXCR4 in blood neutrophils determined by flow cytometry. (n, n = 3-6, one technical replicate of 3-6 biological replicates for each group). Data are mean ± SD. P values are determined by one-way ANOVA. n.s., not significant. Source data for b-h and j-q are provided as a Source Data file.

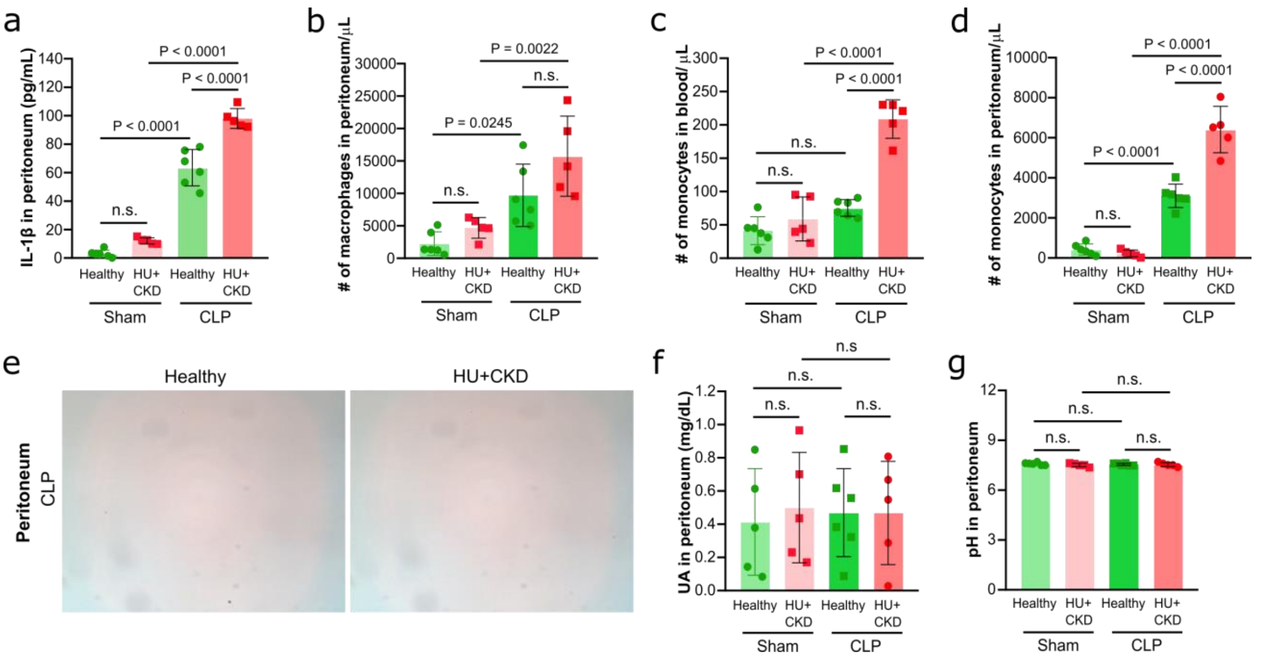

Supplemental Figure S5: UA crystals are not responsible for inducing hyperinflammation in the peritoneum during CLP-induced sepsis. Alb-creERT2;*Glut9*$^{lox/lox}$ mice and *Glut9*$^{lox/lox}$ control mice were injected intraperitoneally with tamoxifen. Both groups were fed either an acidogenic diet with inosine or an inosine-rich diet for 24 days. On day 23, mice underwent either cecal ligation and puncture (CLP) or sham surgery and were sacrificed 24 hours later. (**a**) Concentrations of IL-1β measured in peritoneum from healthy and hyperuricemic CKD (HU+CKD) mice after CLP or sham surgery via ELISA on day 24 (n = 5-6, one technical replicate of 5-6 biological replicates for each group). (**b** - **d**) Number (#) of macrophages in peritoneum (b), and monocytes in blood (c) and peritoneum (d) per µL from healthy and hyperuricemic CKD (HU+CKD) mice after CLP or sham surgery on day 24 determined by flow cytometry. (b-d, n =5-6, one technical replicate of 5-6 biological replicates for each group). (**e**) Representative images of peritoneal wash in healthy and hyperuricemic CKD mice after CLP surgery (original magnification 20x). (**f** and **g**) Peritoneum UA levels (f) and pH (g) in healthy and hyperuricemic CKD mice after CLP or sham surgery on day 24. (f-g, n =5, one technical replicate of 5 biological replicates for each group). Data are mean ± SD. P values are determined by one-way ANOVA. n.s., not significant. Source data for a-d and f-g are provided as a Source Data file.

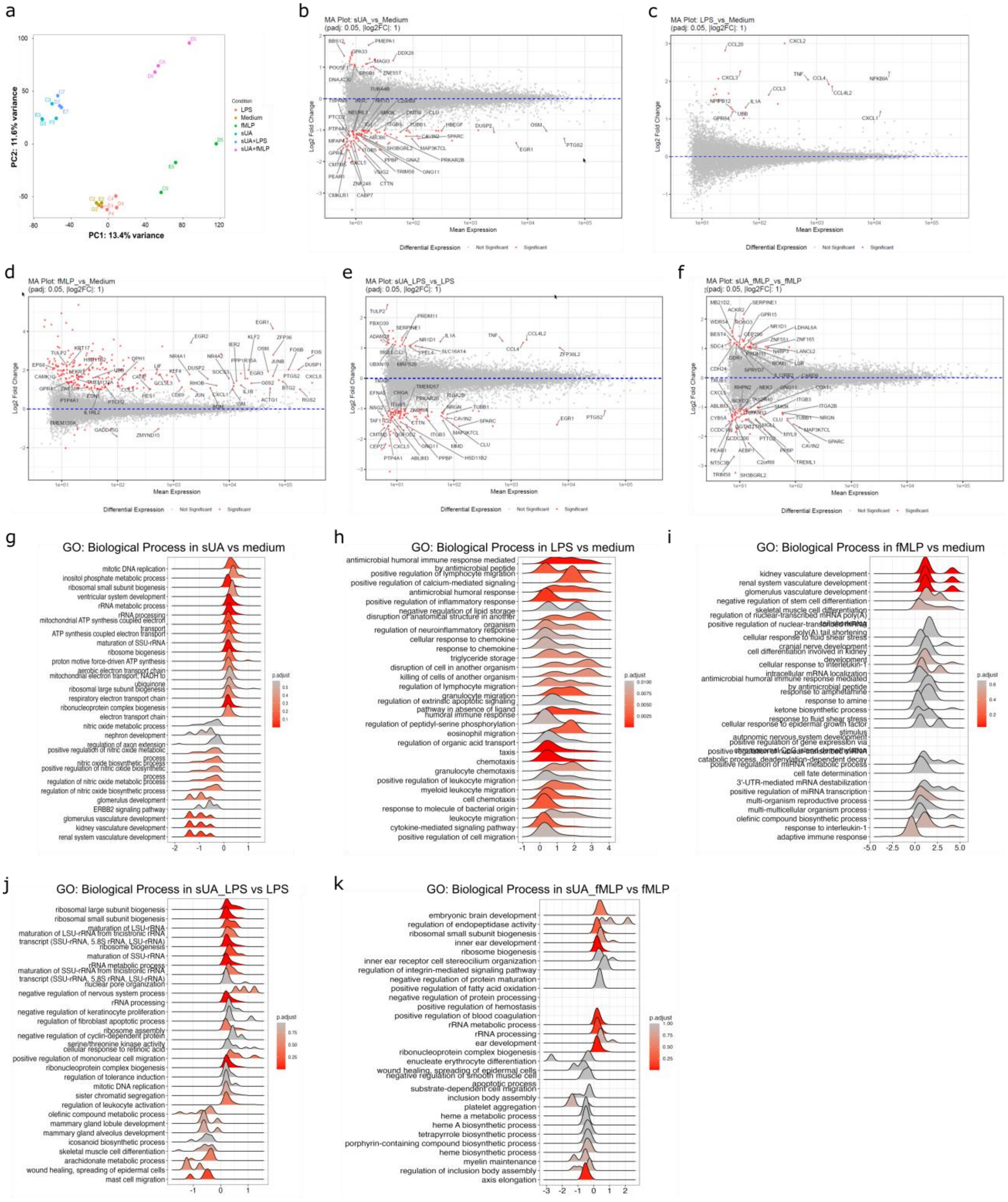

Supplemental Figure S6: Bulk RNA-seq of human neutrophils upon UA, LPS and fMLP stimulation. (**a**) Principal-component analysis (PCA) plot of bulk RNA-seq datasets of human blood neutrophils from healthy individuals stimulated with sUA (n = 3), LPS (n = 4), fMLP (n = 3), sUA+LPS (n = 3), sUA+fMLP (n = 3) or medium (n = 3). Samples are color based on the condition. (a, n = 3, one technical replicate of 3 biological replicates for each group). (**b** to **f**) MA plots showing DEGs in stimulated human neutrophils. Results were ranked and then filtered by

adjusted p-value over 0.1, raw p-value over 0.05 and log2(FC) over 0.1. Legends highlight significantly (red) and not significantly (grey) expressed genes (Methods). (b-f, n = 3, one technical replicate of 3 biological replicates for each group). (**g** to **k**) Gene set enrichment analysis (GSE) was performed and visualized via fgsea and clusterProfiler packages in R with default settings, plotting top 30 categories based on adjusted p-values against multiple functional annotation databases, including Gene Ontology (GO) of biological processes. Ridgeplots show genes as fold change where negative means down-regulated and positive means up-regulated genes. The following comparison pairs are shown: sUA vs medium (g), LPS vs medium (h), fMLP vs medium (i), sUA_LPS vs LPS (j) and sUA_fMLP vs fMLP (k). (g-k, n = 3, one technical replicate of 3 biological replicates for each group).

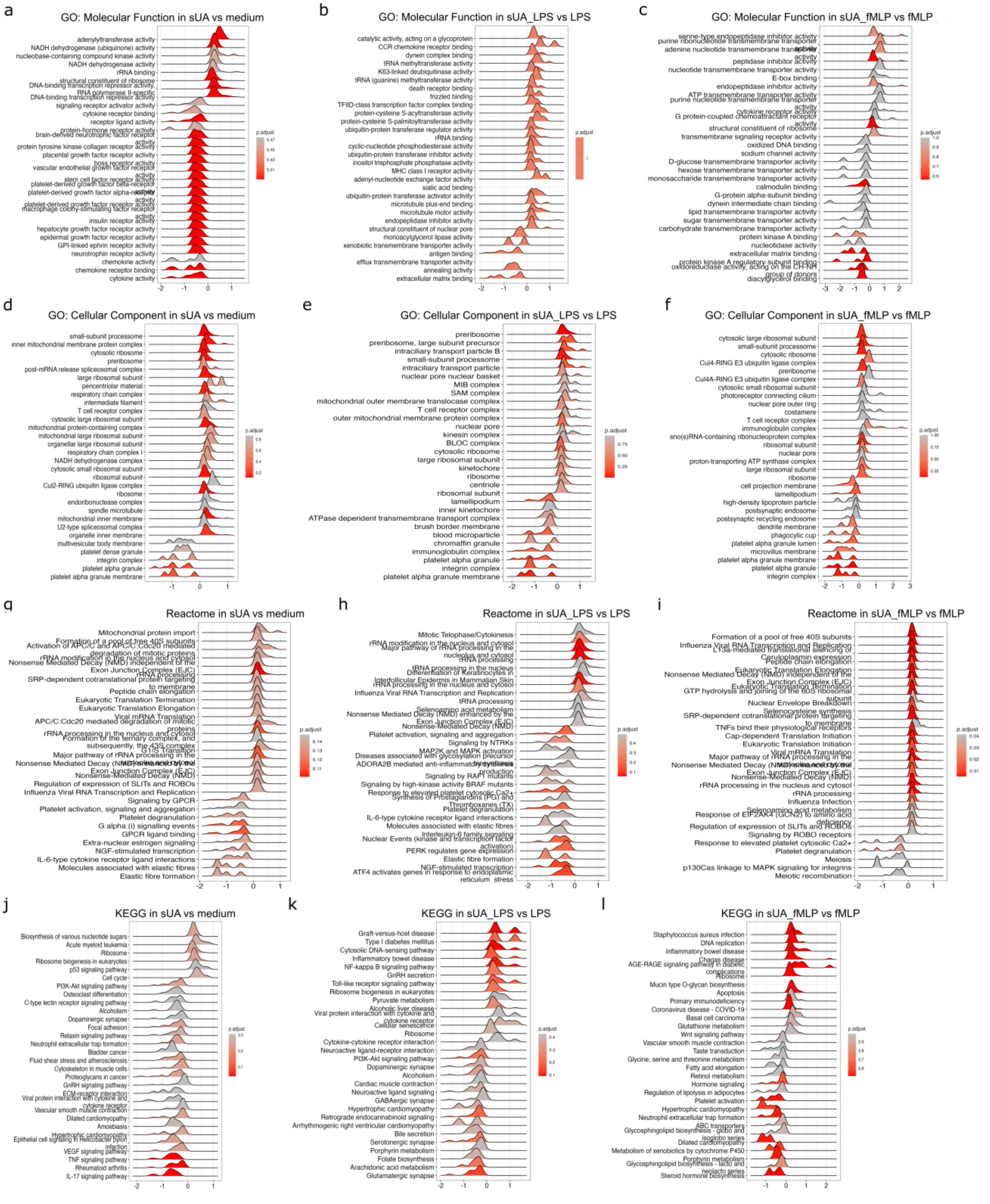

Supplemental Figure S7: Bulk RNA-seq of human neutrophils upon UA, LPS and fMLP stimulation. (**a** to **l**) Gene set enrichment analysis (GSE) was performed of bulk RNA-seq datasets of human blood neutrophils from healthy individuals stimulated with sUA (n = 3), LPS (n = 4), fMLP (n = 3), sUA+LPS (n = 3), sUA+fMLP (n = 3) or medium (n = 3). GSE was visualized via fgsea and clusterProfiler packages in R with default settings, plotting top 30 categories based on

adjusted p-values against multiple functional annotation databases, including Gene Ontology (GO) of moleculare function (a to c) and cellular components (d to f), as well as reactrome analysis (g to i) and KEGG pathway analysis (j to l). Ridgeplots show genes as fold change where negative means down-regulated and positive means up-regulated genes. The following comparison pairs are shown: sUA vs medium (a, d, g, j), sUA_LPS vs LPS (b, e, h, k), and sUA_fMLP vs fMLP (c, f, i, l). (a-l, n = 3, one technical replicate of 3 biological replicates for each group).

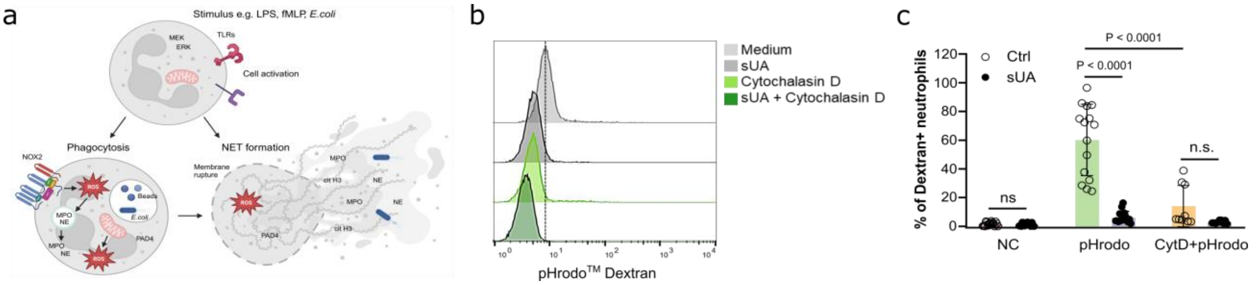

Supplemental Figure S8: Soluble uric acid impairs the ability of human neutrophils to endocytose small particles. (**a**) Schematic of neutrophil signalling pathways and functions in response to stimuli such as LPS, fMLP, and *E.coli,* including phagocytosis/endocytosis, ROS production and NET formation. Created in BioRender. Steiger, S. (2026). https://BioRender.com/tq4hfcl. (**b** and **c**) Endocytosis was measured using pHrodo^{TM} dextran after healthy human blood neutrophils were treated with or without sUA (10 mg/dL) for 30 minutes, followed by incubation with cytochalasin D (CytD, 10 µM) for 10 minutes. Histogram (b) and the percentage (%, c) of endocytosed dextran was determined by flow cytometry (c, n = 16, four technical replicate of 4 biological replicates for each group). Data are mean ± SD. P values are determined by two-way ANOVA. n.s., not significant. Source data for c are provided as a Source Data file.

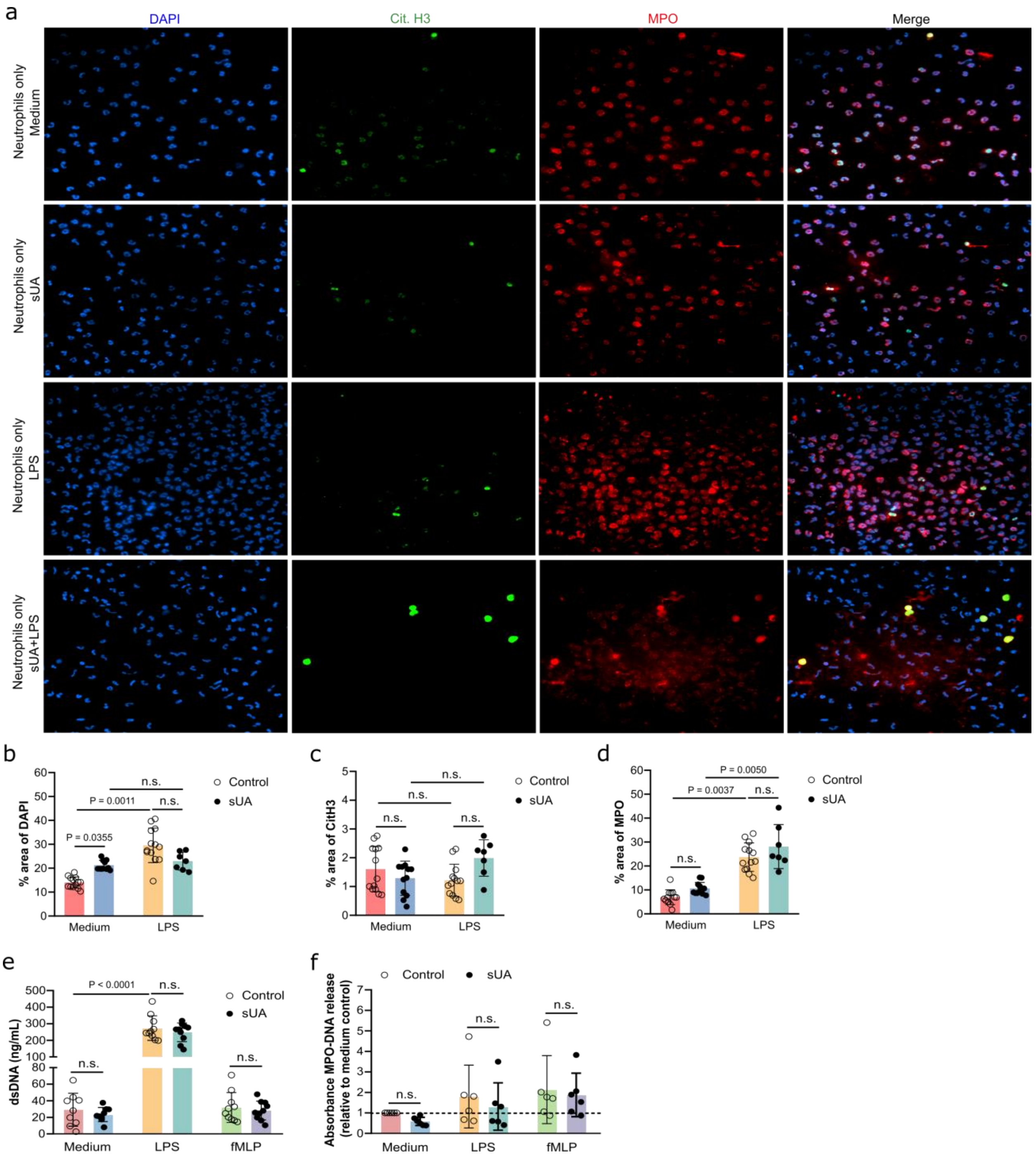

Supplemental Figure S9: Soluble UA does not alter neutrophil extracellular trap formation in LPS-activated human neutrophils. (**a**) Immunofluorescence staining of neutrophil extracellular traps of healthy human blood neutrophils incubated with or without sUA (10 mg/dL) for 30 minutes, followed by incubation with LPS for 30 minutes. NETs were stained for myeloperoxidase (MPO, red), DAPI (blue), and CitH3 (citrullinated histone H3, green). 10x magnification. (**b** – **d**) Quantification of NETs illustrated as percentage of positive area of DAPI (b), CitH3 (c) and MPO (d) using ImageJ. (b-d, n = 8-12, two or three technical replicates of 4-6 biological replicates for each group). (**e** and **f**) NETs were induced with LPS (10 µg/mL) and fMLP (500 ng/mL) for 3 hours

and release of dsDNA (e, ng/mL) (n = 10, duplicates of 5 biological replicates for each group) or MPO-DNA (f, n = 6, one technical replicate of 6 biological replicates for each group) measured in supernatants. Data are mean ± SD. P values are determined by two-way ANOVA. n.s., not significant. Source data for b-f are provided as a Source Data file.

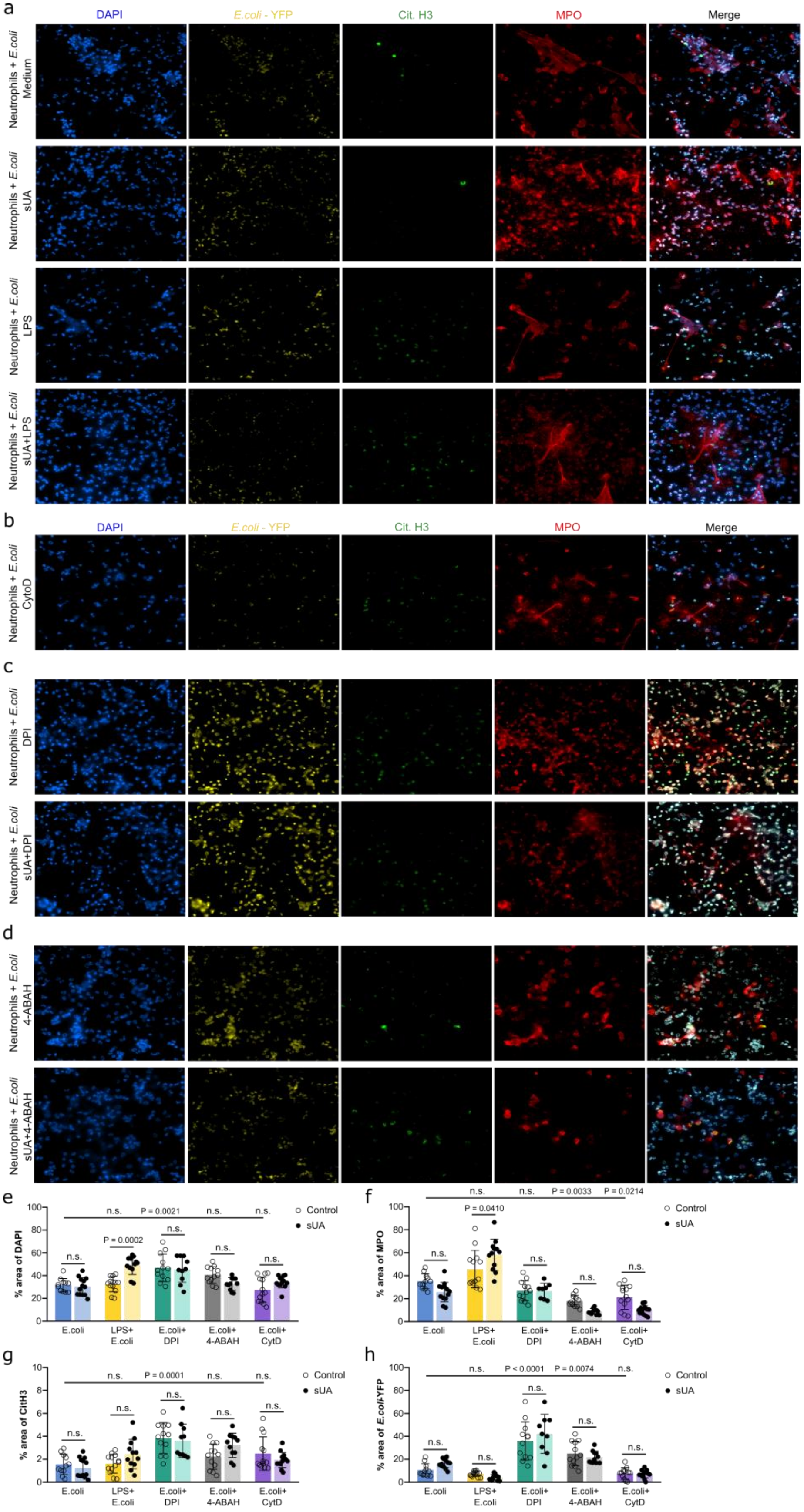
a
DAPI
E.coli - YFP
Cit. H3
MPO
Merge
Neutrophils + E.coli Medium
Neutrophils + E.coli sUA
Neutrophils + E.coli LPS
Neutrophils + E.coli sUA+LPS
b
Neutrophils + E.coli CytoD
c
Neutrophils + E.coli DPI
Neutrophils + E.coli sUA+DPI
d
Neutrophils + E.coli 4-ABAH
Neutrophils + E.coli sUA+4-ABAH
e
% area of DAPI
P = 0.0021
P = 0.0002
n.s.
Control
sUA
E.coli
LPS+ E.coli
E.coli+ DPI
E.coli+ 4-ABAH
E.coli+ CytD
f
% area of MPO
P = 0.0033
P = 0.0214
P = 0.0410
g
% area of CitH3
P = 0.0001
h
% area of E.coli-YFP
P < 0.0001
P = 0.0074

Supplemental Figure S10: Soluble UA does not alter neutrophil extracellular trap formation in *E.coli*-stimulated neutrophils. (**a**) Immunofluorescence staining of neutrophil extracellular traps (NETs) of healthy human blood neutrophils incubated with or without sUA (10 mg/dL) for 30 minutes, followed by incubation with LPS for 30 minutes and co-culture with *E.coli*-YFP at an MOI of 1/15 for 2 hours. NETs were stained for myeloperoxidase (MPO, red), DAPI (blue), and CitH3 (citrullinated histone H3, green). 10x magnification. (**b** to **d**) Human blood neutrophils from healthy individuals were incubated with or without sUA (10 mg/dL) for 30 minutes, followed by incubation with Cytochalasin D (CytoD, 10 µM, b), DPI (50 µM, c), or 4-ABAH (500 µM, d) for 30 minutes. After stimulation, *E.coli*-YFP (yellow) was added for 30 minutes and immunofluorescence staining for NETs performed using myeloperoxidase (MPO, red), DAPI (blue), and CitH3 (citrullinated histone H3, green). 10x magnification. (**e** to **h**) Fluorescence images were quantified as percent (%) area of DAPI (E), MPO (f), CitH3 (g), and *E.coli*-YFP (h) using ImageJ. (e-h, n = 12, three technical replicates of 4 biological replicates for each group). Data are mean ± SD. P values are determined by two-way ANOVA. n.s., not significant. Source data for e-h are provided as a Source Data file.

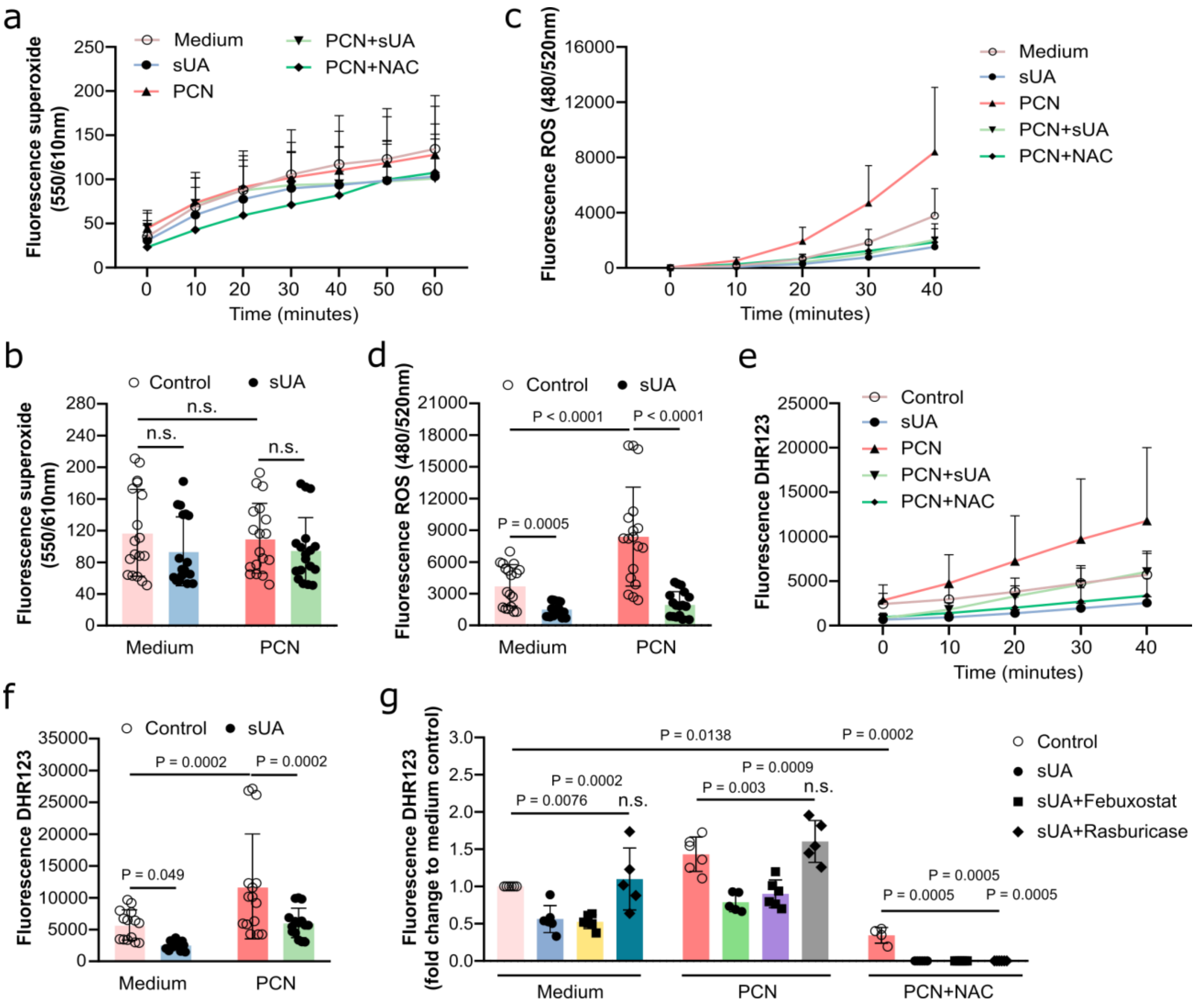

Supplemental Figure S11: Reduced ROS production in human neutrophils following pyocyanin activation. (**a** to **f**) Human blood neutrophils from healthy individuals were pre-incubated with or without sUA (10 mg/dL) or N-acetyl-L-cysteine (NAC, 10 mM) for 30 minutes and stimulated with pyocyanin (PCN, 50 µM). The fluorescence of (**a** and **b**) superoxide (Ex/Em 550/610 nm), (**c** and **d**) total ROS production (Ex/Em 480/520 nm) and (**e** and **f**) intracellular mitochondrial ROS production (DHR 123 at Ex/Em 507/529 nm) was measured over time with an ELISA reader. (a-f, n= 18, triplicates of 6 biological replicates for each group). (**b**, **d**, **f**) representing the intensities at time point 40 minutes. (**g**) Human blood neutrophils from healthy individuals were pre-incubated with or without sUA (10 mg/dL) or febuxostat (50 µM) and rasburicase (1 µg/mL), as well as with NAC (10 mM) for 30 minutes and stimulated with PCN (50 µM). The fluorescence of intracellular mitochondrial ROS production (DHR 123 at Ex/Em 507/529 nm) was measured after 40 minutes with an ELISA reader. (g, n= 5-6, one technical replicate of 6 biological replicates for each group). Data are mean ± SD. P values are determined by two-way ANOVA. n.s., not significant. Source data for a-g are provided as a Source Data file.
