# Supplementary material for: Soluble uric acid suppresses neutrophil-mediated host defense in sepsis
Source: Nat Commun. 2026 May 19;17:4453. doi: 10.1038/s41467-026-73090-4 (PMC13187337; doi:10.1038/s41467-026-73090-4)
Supplement: Supplementary file 2 — Reporting summary [file 41467_2026_73090_MOESM2_ESM.pdf]

nature portfolio

Corresponding author(s): Stefanie Steiger

Last updated by author(s): 07.04.2026

Reporting Summary

Nature Portfolio wishes to improve the reproducibility of the work that we publish. This form provides structure for consistency and transparency in reporting. For further information on Nature Portfolio policies, see our Editorial Policies and the Editorial Policy Checklist.

Statistics

For all statistical analyses, confirm that the following items are present in the figure legend, table legend, main text, or Methods section.

n/a	Confirmed	
☐	☒	The exact sample size (*n*) for each experimental group/condition, given as a discrete number and unit of measurement
☐	☒	A statement on whether measurements were taken from distinct samples or whether the same sample was measured repeatedly
☐	☒	The statistical test(s) used AND whether they are one- or two-sided<br>*Only common tests should be described solely by name; describe more complex techniques in the Methods section.*
☐	☒	A description of all covariates tested
☐	☒	A description of any assumptions or corrections, such as tests of normality and adjustment for multiple comparisons
☐	☒	A full description of the statistical parameters including central tendency (e.g. means) or other basic estimates (e.g. regression coefficient) AND variation (e.g. standard deviation) or associated estimates of uncertainty (e.g. confidence intervals)
☐	☒	For null hypothesis testing, the test statistic (e.g. *F*, *t*, *r*) with confidence intervals, effect sizes, degrees of freedom and *P* value noted<br>*Give P values as exact values whenever suitable.*
☒	☐	For Bayesian analysis, information on the choice of priors and Markov chain Monte Carlo settings
☒	☐	For hierarchical and complex designs, identification of the appropriate level for tests and full reporting of outcomes
☒	☐	Estimates of effect sizes (e.g. Cohen's *d*, Pearson's *r*), indicating how they were calculated

Our web collection on statistics for biologists contains articles on many of the points above.

Software and code

Policy information about availability of computer code

Data collection	Flow Cytometry (BD FACSCalibur and Beckman Coulter CytoFlex), Image J, Multiskan EX reader, Infinite 200 PRO Tecan, Nikon Inverted Research Microscope ECLIPSE Ti2-E, Leica Microscope DMRB 301-371.011
Data analysis	GraphPad Prism 8.0, Image J, FlowJo 8.7, MB Lab software

For manuscripts utilizing custom algorithms or software that are central to the research but not yet described in published literature, software must be made available to editors and reviewers. We strongly encourage code deposition in a community repository (e.g. GitHub). See the Nature Portfolio guidelines for submitting code & software for further information.

Data

Policy information about availability of data

All manuscripts must include a data availability statement. This statement should provide the following information, where applicable:

- Accession codes, unique identifiers, or web links for publicly available datasets
- A description of any restrictions on data availability
- For clinical datasets or third party data, please ensure that the statement adheres to our policy

The Gene Expression Omnibus accession number for the RNA-seq data is GSE294890. Materials, reagents or other experimental data are available upon request.

Research involving human participants, their data, or biological material

Policy information about studies with human participants or human data. See also policy information about sex, gender (identity/presentation), and sexual orientation and race, ethnicity and racism.

Reporting on sex and gender	Both female and male participants were included for statistical analysis.
Reporting on race, ethnicity, or other socially relevant groupings	n/a
Population characteristics	Blood samples from healthy individuals and patients with kidney disease were collected from both sex aged above 18 years or for comparison were aged matched.
Recruitment	All participants voluntarily participated in the study without any self-selection bias and provided informed consent.
Ethics oversight	The study was approved by the local ethical review board of the medical faculty at the Ludwig-Maximilians-University (LMU) Munich (ref: 21-0532) and carried out in accordance with the Declaration of Helsinki for medical research.

Note that full information on the approval of the study protocol must also be provided in the manuscript.

Field-specific reporting

Please select the one below that is the best fit for your research. If you are not sure, read the appropriate sections before making your selection.

☒ Life sciences ☐ Behavioural & social sciences ☐ Ecological, evolutionary & environmental sciences

For a reference copy of the document with all sections, see nature.com/documents/nr-reporting-summary-flat.pdf

Life sciences study design

All studies must disclose on these points even when the disclosure is negative.

Sample size	Samples sizes were chosen to ensure the possibility of statistical analysis and to simultaneously minimize the use of animals in accordance with the animal care guidelines (ARRIVE).
Data exclusions	No data were excluded from the study.
Replication	All animal experimental findings were reproduced as biological replicates at the value stated in figure legends and in vitro experiments were performed with at least 4-10 biological replicates. All results are reproducible.
Randomization	All samples(participants and research animals were randomly assigned to groups.
Blinding	Investigators were blinded to group allocation during data collection. All measurements and analyses were objective.

Reporting for specific materials, systems and methods

We require information from authors about some types of materials, experimental systems and methods used in many studies. Here, indicate whether each material, system or method listed is relevant to your study. If you are not sure if a list item applies to your research, read the appropriate section before selecting a response.

Materials & experimental systems

n/a	Involved in the study	
☐	☒	Antibodies
☒	☐	Eukaryotic cell lines
☒	☐	Palaeontology and archaeology
☐	☒	Animals and other organisms
☒	☐	Clinical data
☒	☐	Dual use research of concern
☒	☐	Plants

Methods

n/a	Involved in the study	
☒	☐	ChIP-seq
☐	☒	Flow cytometry
☒	☐	MRI-based neuroimaging

Antibodies

Antibodies used	Please see method section.
Validation	The antibodies are from commercial sources including BioLegend and information can be found on the manufacturers website.

Animals and other research organisms

Policy information about studies involving animals; ARRIVE guidelines recommended for reporting animal research, and Sex and Gender in Research

Laboratory animals	Eight-week old male and female Alb-creERT2;Glut9lox/lox and Glut9lox/lox mice were used. All mice were bred at the mouse facility at the LMU Munich and housed in groups of 5 in filter-top cages and had access to food and water. Cages, nest lets, food, and water were sterilized by autoclaving before use.
Wild animals	None.
Reporting on sex	Both female and male mice were used.
Field-collected samples	No field-collected samples were used in the study.
Ethics oversight	All animal experiments were performed in accordance with the European protection law of animal welfare and upon approval by the local government authorities Regierung von Oberbayern (ref: ROB-55.2-2532.Vet_02-21-142) based on the European Union directive for the Protection of Animals Used for Scientific Purposes (2010/63/EU) and reported according to the Animal Research: Reporting of In Vivo Experiments (ARRIVE) guidelines

Note that full information on the approval of the study protocol must also be provided in the manuscript.

Plants

Seed stocks	n/a
Novel plant genotypes	n/a
Authentication	n/a

Flow Cytometry

Plots

Confirm that:

☒ The axis labels state the marker and fluorochrome used (e.g. CD4-FITC).

☒ The axis scales are clearly visible. Include numbers along axes only for bottom left plot of group (a 'group' is an analysis of identical markers).

☒ All plots are contour plots with outliers or pseudocolor plots.

☒ A numerical value for number of cells or percentage (with statistics) is provided.

Methodology

Sample preparation	Detailed information on murine and human sample preparation for flow cytometry is provided in the method section.
Instrument	BD FACSCalibur and Beckman Coulter CytoFlex
Software	FlowJo 8.7
Cell population abundance	Human blood neutrophils have a purity of 80-90% after dextran sedimentation.
Gating strategy	See Figures 1e, 2h, and 3j.

☐ Tick this box to confirm that a figure exemplifying the gating strategy is provided in the Supplementary Information.
